# Chiral Perturbation Strategies for Circularly Polarized Thermally Activated Delayed-Fluorescence Small Molecules: Progress in the Application of Organic Light-Emitting Diodes

**DOI:** 10.3390/nano15131053

**Published:** 2025-07-07

**Authors:** Tianwen Fan, Linxian Xu, Hao Tang, Lingyun Wang, Derong Cao

**Affiliations:** 1 State Key Laboratory of Luminescent Materials and Devices, School of Chemistry and Chemical Engineering, South China University of Technology, Guangzhou 510640, China; 202210184856@mail.scut.edu.cn; 2 School of Pharmacy, Guangdong Medical University, Dongguan 523808, China; 3 School of Chemistry and Chemical Engineering, South China University of Technology, Guangzhou 510640, China; haotang@scut.edu.cn (H.T.); lingyun@scut.edu.cn (L.W.)

**Keywords:** thermally activated delayed fluorescence, chirality perturbation, circularly polarized luminescence, light-emitting diode, molecular structure

## Abstract

The application of organic light-emitting diodes (OLEDs) has become widespread, with polarizers commonly employed to mitigate the influence of external light sources on OLED displays. However, when the light signal generated by the OLED emissive layer passes through the polarizer, approximately 50% of the light energy is inevitably lost. Circularly polarized luminescent (CPL) molecules, capable of emitting specific left- or right-handed circularly polarized light, theoretically enable 100% light energy utilization in corresponding OLED devices (CP-OLEDs). With this breakthrough, CPL mechanisms exhibit significant potential for applications in data storage, bioimaging, and 3D displays. In this review, we focus on molecules constructed via a chiral perturbation strategy, analyzing their CPL generation mechanisms and molecular engineering principles. The relationship between these molecular structures and OLED performance is systematically analyzed and summarized. Finally, we critically address current challenges in developing both CPL active materials and devices based on the chiral perturbation strategies, while providing perspectives on future developments and potential challenges in this field.

## 1. Introduction

The rapid evolution of modern technology has exposed critical limitations in conventional display systems, particularly cathode ray tube (CRT) and liquid crystal display (LCD) technologies, rendering them increasingly inadequate for modern applications. This technological gap has created an urgent demand for innovative display solutions capable of meeting escalating societal requirements. Organic light-emitting diodes (OLEDs) have garnered significant attention from both the scientific and business communities due to their advantages, including energy efficiency, portable nature, low power requirements, and high luminance [1,2,3,4,5]. In 1987, Deng, VanSlyke and their colleagues developed the first practical OLED material, significantly propelling the advancement of OLED technology [6]. Subsequent decades of research have witnessed the systematic evolution of OLED emissive materials through three distinct generations: fluorescent materials, phosphorescent materials, and thermally activated delayed-fluorescence (TADF) materials utilizing reverse intersystem crossing mechanisms [7,8,9,10,11,12,13,14].

**Figure 1:**
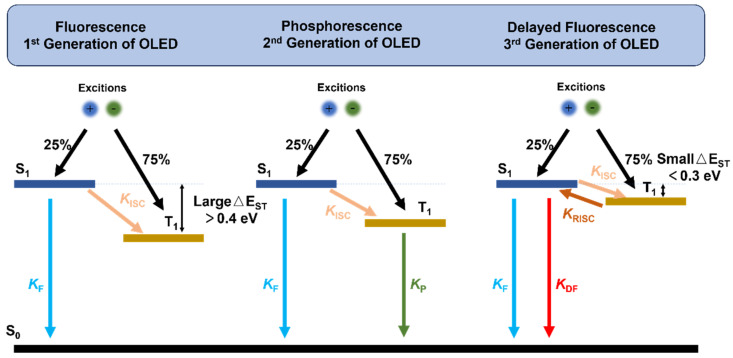
Three generations of OLED emitting material luminescence mechanism. (F = fluorescence; P = phosphorescence; PF = prompt fluorescence; DF = delayed fluorescence; ISC = intersystem crossing; RISC = reverse intersystem crossing; ΔE_ST_ = the energy difference between the first excited singlet and triplet states; nr = nonradiative).

In the first generation of OLED technology, fluorescent materials were employed as the emissive layer. It is well established that fluorescent materials absorb energy and convert it into a 25% singlet state (S_1_) and 75% triplet state (T_1_) (Figure 1). However, only 25% of singlet excitons can return to the ground state via luminescence, while the remaining 75% of triplet excitons are lost through non-radiative transitions. Consequently, the theoretical internal quantum efficiency (IQE) of first-generation OLEDs is limited to 25%, and the maximum external quantum efficiency (EQE) in practical applications is only 5% [3] These limitations restrict first-generation OLEDs from meeting industrial-scale manufacturing requirements and high-performance application standards, thereby driving the imperative for advanced material development. In response to this challenge, scientists discovered that the strategic incorporation of heavy-metal complexes (e.g., Ir(III)- or Ru(II)-based phosphine complexes) could significantly enhance the efficiency of electroluminescence. This improvement is primarily attributed to the spin–orbit coupling effect between organic molecules and heavy-metal centers, which facilitates intersystem crossing (ISC) between the S_1_ and T_1_ states. This enables both singlet and triplet excitons to contribute to light emission, theoretically achieving 100% exciton utilization. Consequently, these phosphorescent systems incorporating heavy-metal centers became the cornerstone of second-generation OLED technology, with commercial devices. Nevertheless, second-generation OLED materials also face critical challenges: prohibitive costs of rare heavy metals, potential environmental pollution, poor material stability, and severe efficiency roll-off. To overcome these fundamental limitations, the third generation of OLED materials, thermally activated delayed-fluorescence (TADF) materials, was introduced by Professor Chihaya Adachi et al. in Japan in 2012 [15]. These materials are designed with a novel molecular structure that minimizes the energy gap between the singlet (S_1_) and triplet (T_1_) states, enabling reverse intersystem crossing (RISC). This process allows triplet excitons to convert into singlet excitons, which emit light through delayed fluorescence. TADF materials theoretically achieve 100% IQE without relying on heavy metals, making them a promising candidate for next-generation OLED emitters [16].

Sustained research efforts in TADF materials have yielded compounds with circularly polarized luminescence (CPL) characteristics, enabling the emission of specific left- or right-handed circularly polarized light. The CPL properties confer critical advantages for display technologies: (1) enhanced image contrast ratio through polarization filtering effects in OLED displays and (2) reduced eye strain via the suppression of parasitic glare [17,18,19,20]. Additionally, the use of metal substrates in OLED devices induces mirror radiation, which interferes with the OLED light source and impacts device performance.

**Figure 2:**
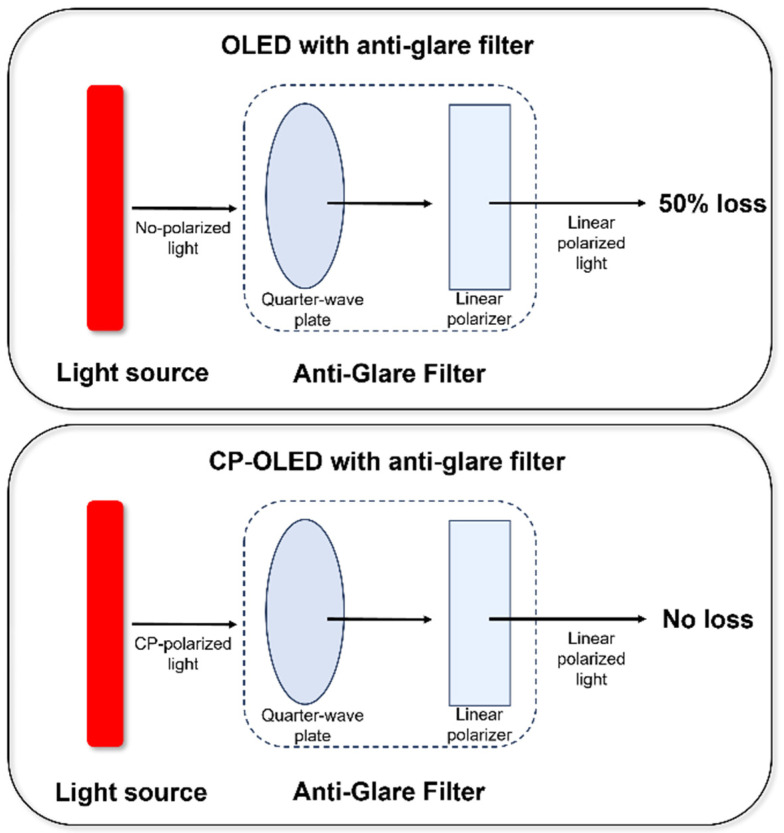
Schematic diagram of an OLED and CP-OLED with anti-glare filters.

In OLED devices, the incorporation of a polarizer is essential to mitigate the effects of external light interference. A typical polarizer consists of a 90° linear polarizer and a 45° quarter-wave plate (QWP) (Figure 2). When external natural light first passes through the 90° linear polarizer, it becomes linearly polarized. Subsequently, it passes through the 45° quarter-wave plate, forming 135° circularly polarized light. When the OLED emits light, it is transmitted through the 45° quarter-wave plate, converting it into 180° linearly polarized light. This emitted light reflects off the OLED electrode and is perpendicular to the 90° linear polarizer, preventing it from passing through. As a result, the reflected light is effectively eliminated, addressing the issue of mirror radiation interference.

However, the use of a polarizer imposes a 50% transmittance penalty on the emitted light intensity, requiring elevated drive current to maintain luminance levels. This, in turn, reduces the operational lifetime of the OLED device. To resolve this limitation, a kind of chiral TADF material (called circularly polarized thermally activated delayed-fluorescent material, CP-TADF), capable of emitting specific left- or right-handed circularly polarized light, has emerged as a promising solution. These materials enable 100% of the emitted light to pass through the polarizer, fundamentally overcoming the issue of energy loss. OLED devices based on CP-TADF materials are referred to as circularly polarized light-emitting diodes (CP-OLEDs) and are considered to be the most promising candidates for next-generation light-emitting materials.

CP-TADF materials encompass both small molecules and polymers [21,22,23,24]. Two primary molecular design strategies are employed. The first strategy involves luminescent molecules that possess inherent chiral characteristics, such as helicene, naphthoquinone, and [2,2]-paracyclophane. The second strategy utilizes the chiral perturbation strategy, which integrates luminescent molecules with chiral units, such as binaphthol, octahydrobinaphthol, 1,2-diaminocyclohexane, and alkyl chains.

Chiral materials, which possess inherent asymmetry, are capable of producing left or right circularly polarized light with varying intensities as they transition from an excited state back to the ground state. To quantify the intensity of this circularly polarized light, the asymmetry factor (g) is utilized. This factor is calculated using the formula, g = 2 (I_L_-I_R_)/(I_L_ + I_R_), where I_L_ and I_R_ represent the intensities of the left and right circularly polarized light, respectively. To further assess the performance of CP-TADF materials, a comprehensive evaluation formula, *FM*, is introduced. This formula integrates the luminescence asymmetry factor (g_lum_) and the photoluminescence quantum yield (PLQY), expressed as *FM* = g_lum_ × PLQY = 2 (NL − NR)/Na. Here, *NL*, *NR*, and *Na* denote the numbers of emitted left-handed photons, right-handed photons, and absorbed photons, respectively [25]. The design of CP-TADF materials, therefore, must ensure both high PLQY and g_EL_ to optimize their performance.

The development of TADF molecules with circularly polarized luminescence properties was pioneered by Imagawa et al. in 2015 [25]. Since then, the design strategies for CP-TADF materials have undergone significant refinement, leading to the creation of numerous high-performance CP-TADF materials suitable for OLED applications. However, the ongoing challenge lies in the development of chiral TADFs that exhibit both high PLQY and g_EL_. This paper provides a comprehensive review of CP-TADF materials developed through the chiral perturbation strategy since 2015, focusing on their design concepts, luminescent properties, and device applications. The insights presented aim to facilitate the creation of more advanced and high-performance CP-TADF molecules, thereby advancing the field of OLED technology.

## 2. Molecular Design of CP-TADF Materials and Performance of Electroluminescent Devices

### 2.1. Binaphthol

**Figure 3:**
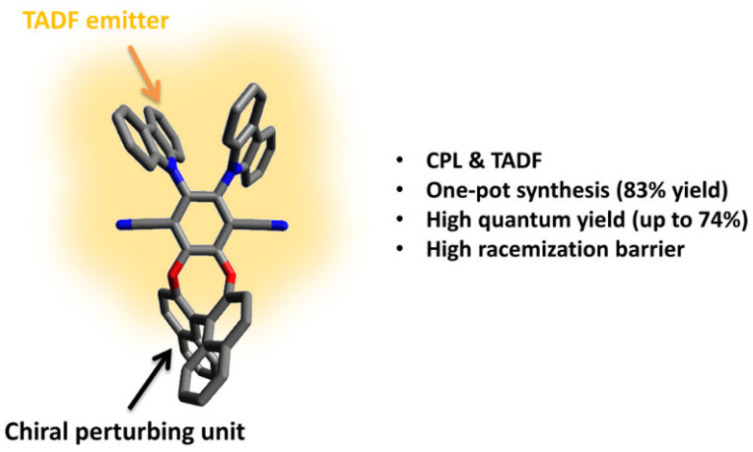
(R/S) – 1 molecular design schematic diagram. Reproduced with permission. Copyright © 2016 American Chemical Society [26].

**Figure 4:**
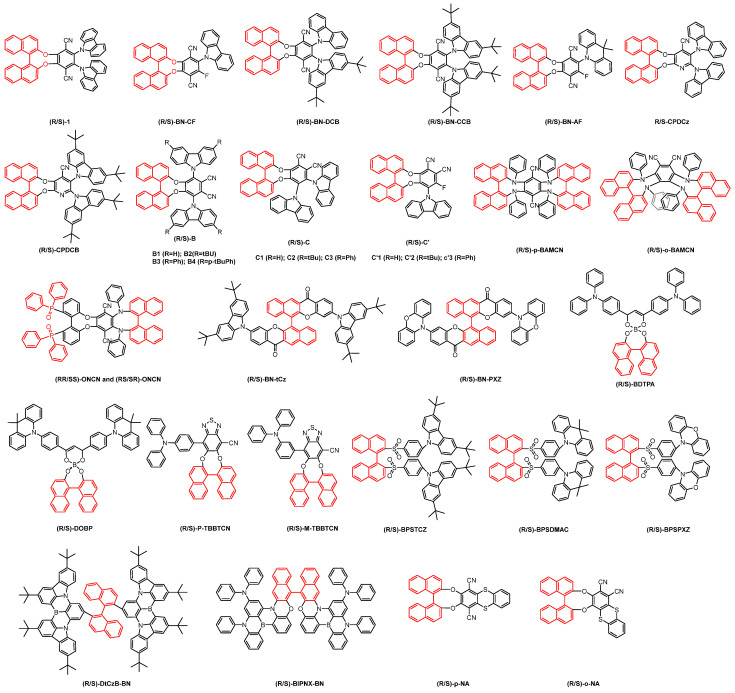
Binaphthol based chiral CP-TADF molecules (The red part in the picture represents the chiral unit).

In 2016, Pieters and colleagues introduced a chiral perturbation strategy, incorporating chiral naphthol as a chiral unit to interface with efficient TADF materials, thereby constructing the CP-TADF material (R/S)-1 (Figure 3). This molecule exhibited a favorable synthetic yield of 84% [26]. In solution, it achieved a quantum yield (QY) of 74%, a delayed-fluorescence lifetime of 2.9 µs, and a notable luminescence asymmetry factor (g_lum_) of 1.3 × 10^−3^. When incorporated into an organic light-emitting diode (OLED) at a 20% doping concentration, the resulting device exhibited a maximum EQE of 9.1%. However, corresponding circularly polarized electroluminescence (CPEL) data was not obtained for this device (Figure 4).

On the basis of previous work, Tang et al. systematically modified molecular structures to develop a series of CP-TADF materials, including (R/S)-BN-CF, (R/S)-BN-AF, (R/S)-BN-CCB, and (R/S)-BN-DCB, by altering the donor and acceptor units [27]. These compounds exhibit outstanding aggregation-induced emission (AIE) and CPL characteristics, with high PLQY and g_lum_ values in both solution and film states. Notably, by maintaining the chiral unit and electron acceptor while simply modifying the donor unit, the electroluminescence band maxima can be tuned across the range of 493–571 nm. When employed as emissive layer materials in OLEDs, these molecules achieve a maximum EQE up to 9.3% in the doped system, along with a high |g_EL_| value of 2.6 × 10^−2^. Due to the small distance between the chiral unit and the TADF skeleton, chirality is well extended to the TADF skeleton, resulting in strong g_EL_ for all four molecules. When the electron donor changes from weakly charged carbazole to strongly charged 9,9-dimethylcarbazine, the luminescent color shows a red shift of around 80 nm. Due to the non-radiative transition enhancement dominated by the Energy Gap Law, the maximum EQE of BN-AF is much smaller than that of BN-CF.

To investigate the influence of receptors in different π systems on material properties, Huang et al. incorporated cyanopyridine as the electron acceptor within the TADF framework, combined with carbazole and carbazole derivatives as electron donors, to synthesize a series of CP-TADF materials (R/S)-CDPCz and (R/S)-CDPCB (Figure 4) [28]. In a THF solution, these molecules exhibited a PLQY of 37%, which increased to 55% when formulated into thin films with the main 1,3-di(9 *H*-carbazol-9-yl)benzen (mCP) at a mass ratio of 10%. The CPDCB device, fabricated via both vapor deposition and solution-based methods, demonstrated a maximum emission peak at 515 nm, achieving a maximum EQE of 12.4% and an electroluminescence asymmetry factor |g_EL_| of 8.6 × 10^−4^. In contrast, the CPDCz-based device exhibited a maximum emission peak at 496 nm, with a maximum EQE of 10.1% and an electroluminescence asymmetry factor |g_EL_| of 3.7 × 10^−3^.

Pieters et al. constructed a series of DATF molecules using carbazole and its derivatives with dicyanobenzene and developed a series of CP-TADF materials—(R/S)-B, (R/S)-C’, and (R/S)-C—in combination with chiral binaphthol. They systematically studied the relationship between the molecular structure and properties of these CP-TADF materials [29]. A novel strategy was proposed to enhance CPL performance by reducing the distance between TADF luminescent groups and chiral units. These materials utilize benzene rings at different cyano positions as acceptor units and carbazole and its derivatives as donor units. Theoretical calculations revealed that the HOMO is predominantly distributed in the donor unit, while the LUMO is localized in the acceptor unit, with chiral binaphthol showing minimal involvement in frontier orbital distribution. The emission peaks of these molecules span the range of 469–519 nm, with PLQY ranging from 2% to 30%. In chiral testing, except for C1 and C2, which exhibit negligible chiral signals, all other molecules demonstrate good chiral performance. Notably, B1 exhibits the best electroluminescence asymmetry factor of 2 × 10^−3^. Additionally, they introduced a top emission structure for the first time, enabling the preparation of the material into an OLED device and significantly enhancing the CPEL signal. This work prepared a series of molecules to verify the significance of the magnetic transition dipole moment (µ_m_) value and the resulting angle *θ* between the electrical dipole moment and the magnetic dipole moment. We need to find an optimal balance between µ_m_ and *θ* in order to obtain a fluorescent material with the best chiral electroluminescence.

In 2021, Zheng et al. developed two pairs of D*-A-D* skeleton CP-TADF materials, namely (R/S)-p-BAMCN (rod-shaped) and (R/S)-o-BAMCN (helical), based on chiral binaphthol (Figure 4) [30]. These materials utilize aniline as the donor and p-cyanobenzene as the acceptor. Due to their rigid molecular structure and unique molecular arrangement, they exhibit excellent TADF performance, with high PLQY of 86% and 77% in toluene. When employed as the emissive layer in OLEDs, these molecules achieve maximum emission peaks at 530 nm and 506 nm, with maximum EQE of 27.6% and 20.5%, respectively. The corresponding CP-OLED device demonstrates an excellent electroluminescence asymmetry factor |g_EL_| of approximately 4.6 × 10^−3^. They further investigated the effects of two different chiral units on chiral properties. Based on their previous work, one of the binaphthols was substituted with a specific chiral unit, leading to the synthesis of two enantiomers: (RR/SS)-ONCN and (RS/SR)-ONCN [31]. The absolute photoluminescence quantum yields of these molecules in the thin film state are 80% and 76%, respectively, and both exhibit TADF characteristics. Through chiral studies, it was found that (RR/SS)-OCN (|g_lum_| = 2.0 × 10^−3^) exhibits stronger circularly polarized luminescence than (RS/SR)-OCN (|g_lum_| = 1.3 × 10^−3^). This indicates a certain stacking effect between the two chiral units that promotes molecular chiral emission. (RR)-ONCN and (SR)-ONCN molecules were prepared into OLED devices, achieving emission peaks at 518 and 528 nm, with maximum EQE values of 20.0% and 21.9%, respectively. Correspondingly, the CP-OLED devices based on (RR/SS)-ONCN and (RS/SR)-ONCN exhibited excellent electrochromic chiral luminescence performance, with |g_EL_| factors of 1.1 × 10^−3^ and 0.7 × 10^−3^, respectively.

In order to expand the emission color range of CP-OLED molecules, Cheng et al. designed two pairs of chiral unit and acceptor molecules based on chiral binaphthol. These molecules were combined with electron donors, tert-butyl carbazole (tCz) and phenoxazine (PXZ), to form new CP-TADF materials: (R/S)-BN-tCz and (R/S)-BN-PXZ [32]. The PLQY increased from 47.8% to 66.9% for BN-tCz and from 47.9% to 68.0% for BN-PXZ, respectively, when transitioning from the solution state to the film state. BN-tCz exhibits blue emission, while BN-PXZ exhibits orange emission. When doped together and processed into OLED devices via a solution-based method, these molecules produce white circularly polarized luminescence with a maximum EQE of 1.0% and a circularly polarized electroluminescence asymmetry factor |g_EL_| of approximately 2 × 10^−3^. This work represents the first report on an efficient white CP-OLED material.

Chen et al. synthesized a pair of enantiomers, (R/S)-BDTPA, by choosing bi-phenyl boron β-diketone acid as the acceptor and diphenylamine as the donor unit based on naphthalene [33]. Due to the direct involvement of chiral units in TADF emission and effective chiral transfer achieved through the intramolecular charge transfer (ICT) effect, the molecule exhibits excellent optical stability, mirror circular dichroism (CD), and CPL activity, with a maximum PLQY of 15.8% in the doped film. Using (R/S)-BDTPA as the emitting layer in OLED devices, a red CP-OLED (λ_EL_ = 600 nm) was successfully fabricated with a maximum EQE of 2.0% and a maximum electroluminescence asymmetry factor |g_EL_| of 2.1 × 10^−3^. Building on this work, Yang et al. changed the electron donor to dimethylacridine to prepare the enantiomer (R/S)-DOBP, which resulted in a significant red shift in the emission wavelength [34]. The highly distorted structure confers the molecule with excellent photophysical properties, and the introduction of naphthol provides excellent chiral properties, with g_lum_ of 2.5 × 10^−4^. The CP-OLED device prepared based on this enantiomer can generate near-infrared light (716 nm), with a maximum external quantum efficiency of 1.9%. Building on this work, Tang et al. modified one of the electron donors by introducing an electron-withdrawing cyanide group and further adjusted the substitution position of triphenylamine. This resulted in the synthesis of two enantiomers: (R/S)-P-TBBTCN and (R/S)-M-TBBTCN [35]. By altering the substitution position of triphenylamine, they achieved not only emission redshift but also enhanced the AIE effect, delayed-fluorescence characteristics, and chiral emission intensity. The g_lum_ of R/S-M-TBBTCN (R: 1.03 × 10^−3^, S: 1.07 × 10^−3^) are much higher than those of R/S-P-TBBTCN (R: 2.18 × 10^−5^, S: 1.16 × 10^−4^). The maximum EQE of the devices fabricated using these enantiomers were 2.08%, 1.60%, 2.19%, and 1.43% for S-P-TBBTCN, R-P-TBBTCN, S-M-TBBTCN, and R-M-TBBTCN, respectively. For red and near-infrared luminescence, due to the low OLED luminous efficiency, the corresponding CPEL signal is relatively low, making it difficult to detect the CPL signal. Improving the luminescence efficiency of red and near-infrared molecules is also an important factor for enhancing glum. For red and near-infrared luminescence, due to the low OLED luminous efficiency, the corresponding CPEL signal is relatively low, making it difficult to detect the CPL signal. Improving the luminescence efficiency of red and near-infrared molecules is also an important factor for enhancing g_lum_.

In 2023, Zheng et al. developed a new sulfonyl naphthalene derivative using naphthol as both an electron acceptor and chiral unit. By integrating this with tert-butylcarbazole, 9,9-dimethyl-9,10-dihydroacridine, and phenoxazine structures, they synthesized three chiral enantiomers: (R/S)-BPSTCZ, (R/S)-BPSDMAC, and (R/S)-BPSPXZ [36]. These materials demonstrated high fluorescence quantum yields in their respective co-doped films, achieving 69%, 74%, and 88%, respectively. When incorporated into OLED devices, the remaining three enantiomers exhibited maximum EQE of 3.8% (λ_EL_ = 438 nm), 7.5% (λ_EL_ = 464 nm), and 28.5% (λ_EL_ = 515 nm). Circular polarized OLED displays excellent CPEL signals, among which BPSPXZ exhibits the best CPEL signal performance due to its better photoluminescence performance and electroluminescence efficiency, achieving an |g_EL_| of 8.8 × 10^−3^.

The unique narrow emission and high EQE characteristics of multiple resonance (MR) materials have been increasingly studied. Introducing chiral units into excellent multiple resonance molecules that have been reported to prepare CP-OLED materials is an extremely efficient approach. Zheng et al. developed a novel approach by integrating naphthol with a boron nitride skeleton, DtCzB, to create enantiomers (R/S)-DtCzB-BN [37]. These materials retain the TADF properties of multiple resonant host molecules, achieving an exceptional PLQY of up to 98% in thin films. The corresponding CP-OLED exhibits a maximum emission peak at 494 nm, with a narrow half-peak width of 22 nm and a high maximum EQE of 33.9%. The g_EL_ values are +7.99 × 10^−4^ and −8.19 × 10^−4^, demonstrating robust circularly polarized light emission. Furthermore, Zheng et al. introduced an innovative method for constructing chiral multiple resonance materials by conjugating and extending chiral biphenyl structures. This approach synthesizes (R/S)-BIPNX-BN, which exhibits outstanding photoluminescence characteristics, including a PLQY of up to 93.7% and a half-peak width of 30 nm [38]. When incorporated into CP-OLED devices, (R/S)-BIPNX-BN achieves a maximum EQE of 24.2% at an emission wavelength of 479 nm, with corresponding g_EL_ values of +1.0 × 10^−3^ and −1.3 × 10^−3^. This research underscores the potential of chiral multiple resonance materials in advancing the performance of CP-OLEDs. By comparing the above two works, it was found that chiral units in the MR skeleton may have a positive effect on improving chiral properties.

The coexistence of TADF and room-temperature phosphorescence (RTP) mechanisms in a molecule has a positive effect on achieving efficient luminescence. Su et al. combined phenyldithiol, naphthol, and dicyanobenzene to form a unique multi-ring donor–acceptor skeleton, resulting in a series of chiral compounds, (R/S)-p-NA and (R/S)-o-NA, with unique luminescent properties (Figure 4) [39]. By altering the positions of the two cyanide groups on the central benzene ring, two molecules with completely different luminescent properties can be obtained. Specifically, (R/S)-p-NA exhibits excellent TADF properties, with a PLQY of 91.4% in the thin film state. It corresponds to a maximum emission peak at 515 nm and a maximum EQE of 29.1%. In contrast, (R/S)-o-NA possesses both TADF and RTP mechanisms. Although its fluorescence quantum yield is only 22.1% in the thin film, the device based on (R/S)-o-NA emits warm white light with a maximum EQE of 2.5%.

### 2.2. Octahydronaphthol

**Figure 5:**
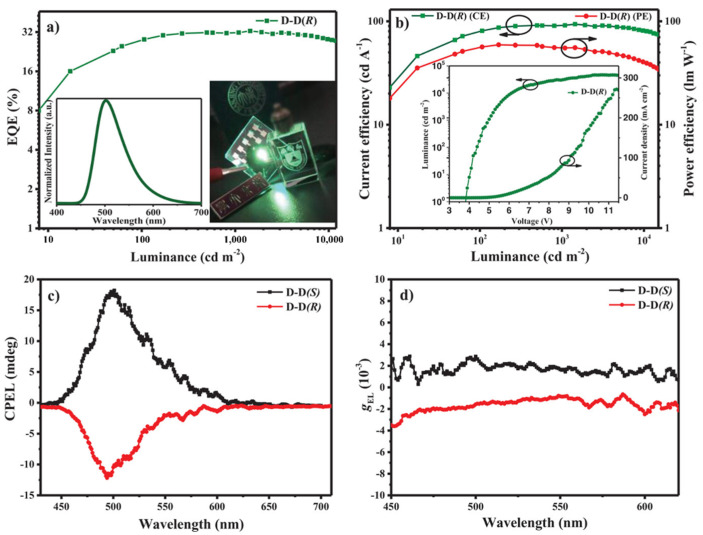
Performance of CP-doped OLED based on (R/S)-OBN-DPA: (**a**) EQE–luminance curves with insertion of EL spectrum at 6 V and lighting of NJU logo by device D-D(R). (**b**) Current–efficiency/power–efficiency–luminance curves and insertion of current–density/luminance–voltage curves. (**c**) CPEL spectra. (**d**) g_EL_ versus wavelength curves. Copyright 2019, Wiley-VCH [41].

In 2019, Zheng et al. first reported an enantiomer (R/S)-OBN-DPA, based on octahydronaphthol as a chiral unit, diphenylamine as an electron donor, and dicyanobenzene as an electron acceptor [40]. The maximum PLQY of this molecule in undoped thin films is 78.59%, while in the 2,6-bis (3-(9 *H*-carbazol-9-yl) phenyl) pyridine doped system, the PLQY is 84.61%. The OLED emitting layer, prepared by vacuum evaporation in a doped system, displayed a maximum emission peak at 544 nm with maximum EQE values of 12.3% and 12.4%, respectively. The electroluminescence asymmetry factors under doping were 2.3 × 10^−3^ and −1.8 × 10^−3^, respectively. Building on this work, Zheng et al. replaced the electron donor with carbazole, further improving the PLQY to 92%. OLED devices fabricated using (R/S)-OBN-Cz as the luminescent layer material exhibited a high maximum EQE of 31.7% in the doped system, with an emission peak at 501 nm [41]. The efficiency roll-off of the OLED was exceptionally low, with roll-offs of 2.5%, 1.0%, and 4.4% at brightness levels of 1000, 3000, and 5000 cd/m^2^, respectively. The corresponding electroluminescence asymmetry factors |g_EL_| were 2.30 × 10^−3^ and 1.94 × 10^−3^ (Figure 5). By simply changing the type of donor from diphenylamine to carbazole, fluorescent materials with better electroluminescence performance can be obtained without affecting g_EL_.

**Figure 6:**
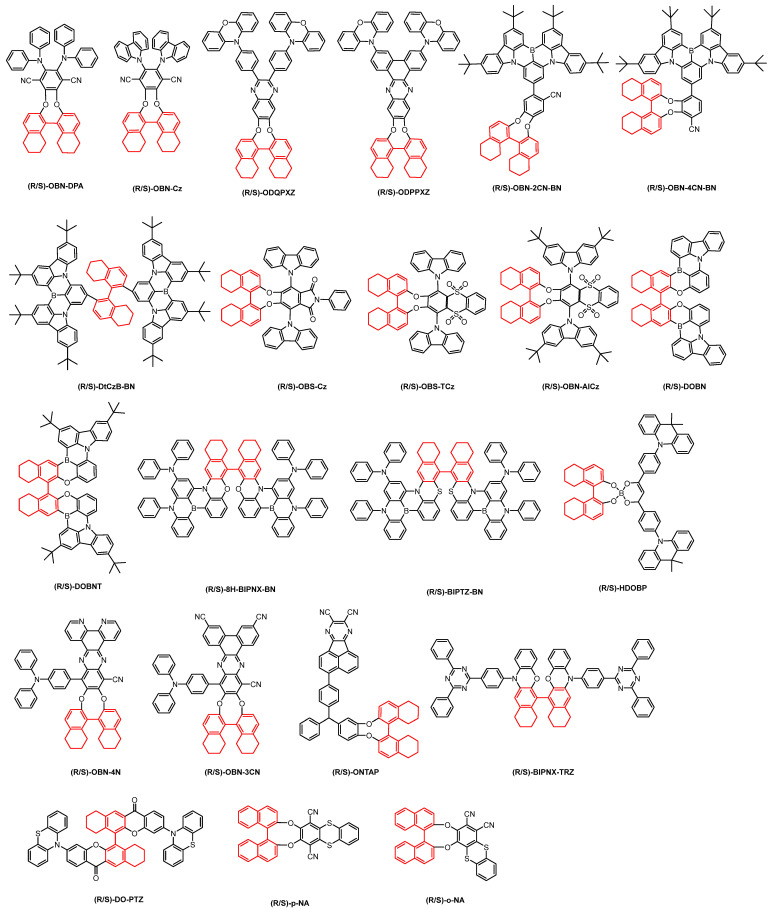
Octahydrobinaphthol-based chiral CP-TADF molecules. (The red part in the picture represents the chiral unit).

In 2021, Tang et al. successfully synthesized two pairs of enantiomeric molecules, (R/S)-ODQPXZ and (R/S)-ODPPXZ (Figure 6), via chiral octahydrobinaphtol, with diphenylquinoxaline as the receptor unit and dibenzophenazine as the donor unit [42]. These molecules exhibit small ΔE_ST_ values of 0.16 and 0.07 eV, accompanied by high PLQY of 92% and 89%, respectively. Devices fabricated using these CP-TADF materials demonstrate outstanding performance, with maximum emission peaks at 548 and 600 nm, maximum EQE values of 28.3% and 20.3%, respectively, and a |g_EL_| of 0.6 × 10^−3^ and 2.4 × 10^−3^. Notably, the larger twist angle and more rigid structure of ODPPXZ contribute to its superior efficiency and enhanced CPL performance.

As research on multiple-resonance materials has advanced, their application in the preparation of CP-OLED light-emitting layers has significantly expanded their scope. In 2021, Li et al. first utilized the multiple-resonance framework DtBuCzB as the donor unit and benzonitrile as the acceptor unit, integrating chiral octahydrobinaphtol to synthesize two pairs of CP-TADF enantiomers: (R/S)-OBN-2CN-BN and (R/S)-OBN-4CN-BN (Figure 6) [43]. These materials exhibit extremely high photoluminescence quantum yields (PLQY) of 99% and 96%, respectively, in toluene solution. When prepared as the luminescent layers of OLEDs, (R/S)-OBN-2CN-BN and (R/S)-OBN-4CN-BN demonstrate small half-widths of 30 and 33 nm, respectively, with maximum EQE values of 29.4% (λ_EL_ = 496 nm) and 24.5% (λ_EL_ = 508 nm), respectively. The electroluminescence asymmetry factor |g_EL_| reaches 1.43 × 10^−3^ and 4.76 × 10^−4^, respectively. Meanwhile, Zheng et al. explored different connection methods and incorporated two multiple-resonance molecules onto a chiral unit to synthesize the enantiomer (R/S)-DTzB-OBN. This material retains the excellent thermally activated delayed-fluorescence (TADF) properties of multiple-resonance molecules and achieves a fluorescence quantum yield of up to 97% in thin films. When used as the light-emitting layer of OLED devices, it exhibits an emission peak at 493 nm with a half-width of only 22 nm and a maximum EQE of 30.0%. The corresponding CP-OLED displays a symmetrical circularly polarized electroluminescence (CPEL) spectrum with |g_EL_| values of 3.40 (±0.362) × 10^−4^ and 3.11 (±0.362) × 10^−4^, respectively. Analysis of the above two works reveals that chiral naphthol has a relatively low impact on the luminescence performance of the molecule, but the number of chiral units and TADF acceptors within the entire molecule has a significant influence on CPEL. When the ratio of chiral units to TADF acceptors is 1:1, the CPEL signal will be better than when it is 1:2.

In 2022, Chen et al. successfully synthesized a pair of CP-TADF molecules, (R/S)-OBN-AICz, utilizing chiral octahydrobinaphtol as the chiral skeleton, aromatic imides as acceptor units, and carbazole as donor units [44]. The molecule’s highly distorted conformation and significant torsion angle between the donor and acceptor units result in efficient TADF properties. Specifically, the molecule exhibits a small energy difference between the singlet and triplet states (ΔE_ST_) of 0.08 eV and a high photoluminescence quantum yield (PLQY) of 81% in doped systems. When implemented as the emitting layer in OLED devices, (R/S)-OBN-AICz achieves a maximum emission peak at 514 nm, with maximum EQE values of 19.0% and 18.7%, respectively. The corresponding CPEL asymmetry factors |g_EL_| are measured as 4.7 × 10^−4^ and 6.5 × 10^−4^. Shortly thereafter, Zheng et al. modified the receptor unit by incorporating highly electronegative 5,5,10,10-tetraoxides (TTR), leading to the synthesis of two pairs of enantiomers: (R/S)-OBS-Cz and (R/S)-OBS-TCz [45]. These molecules utilize carbazole and 3,6-Di-tert-butylcarbazole as the donor units. The resulting PLQY values for these enantiomers are 73% and 87%, respectively. OLED devices fabricated using these materials demonstrate good external quantum efficiency, with maximum EQE values of 15.0% for R-OBS-Cz and 20.3% for R-OBS-TCz. Additionally, these devices exhibit a significant CPEL signal, with electroluminescence asymmetry factors |g_EL_| reaching on the order of 10^−3^.

In 2022, Zheng et al. adopted a steric hindrance-assisted dual-core strategy to construct two pairs of CP-MR-TADF molecules, (R/S)-DOBN and (R/S)-DOBNT, based on octahydrobinaphtol [46]. Both enantiomers exhibit excellent TADF properties, with maximum PLQY of 91% and 93%, respectively. When prepared as the luminescent layer of OLED devices, these molecules exhibit maximum emission peaks around 560 nm with a narrow half-peak width of approximately 35 nm. The corresponding maximum EQE values are 23.9% and 25.6%, respectively. The circularly polarized electroluminescence (CPEL) asymmetry factors |g_EL_| are 0.9 × 10^−3^ and 1.0 × 10^−3^, respectively. Shortly thereafter, they advanced their research and prepared two additional pairs of enantiomers, (R/S)-8*H*-BIPNX-BN and (R/S)-8*H*-BIPTZ-BN [38]. These molecules demonstrate excellent photoluminescence performance, with half-peak widths of 37 nm and 43 nm, respectively. Their fluorescence quantum yields in toluene are 93.2% and 96.1%, respectively. OLED devices based on these materials exhibit narrowed half-peak widths of 30 nm and 28 nm, with maximums of 23.4% (λ_EL_= 482 nm) and 26.3% (λ_EL_ = 487 nm), respectively. The devices also display excellent CPEL characteristics, where the |g_EL_| can reach 1.9 × 10^−4^ and 1.0 × 10^−4^, respectively. The introduction of tertbutyl and heavy atoms has a positive effect on the construction of CP-TADF molecules using a rigid inference-assisted dual core strategy. There is uncertainty about the impact on chiral luminescence performance, but it can improve the maximum EQE of the device.

Due to the narrow bandgap of red and near-infrared molecules, it is challenging to develop efficient red- or near-infrared-emitting OLEDs. In 2022, Yang et al. synthesized a pair of chiral TADF enantiomers, (R/S)-HDOBP (Figure 6), utilizing chiral octahydronaphthol as the chiral skeleton [34]. The acceptor unit was 1,3-bis(4–1,3-bis(4-bromophenyl)-3-hydroxypropyl-2-en-1-one, while the donor unit was 9,9-dimethyl-9,10-dihydroacridine. These molecules not only exhibit excellent TADF properties, but also possess unique mechanical color changes, pressure-induced color changes, and AIE characteristics due to their twisted skeletons. Chiral studies reveal that the pair of molecules demonstrate outstanding circularly polarized luminescence properties, with a luminescence asymmetry factor of ±1.5 × 10^−4^. When employed as the light-emitting layer in OLEDs, these molecules achieve near-infrared emission with emission peaks at 700 nm and a maximum EQE value of 0.7%. Fan et al. developed two chiral enantiomers, (R/S)-OBN-4N and (R/S)-OBN-3CN, by combining polycyclic aromatic chromophores with chiral octahydronaphthol [47]. These molecules exhibit exceptional TADF performance, with PLQYs of 93% and 87% in thin films, respectively. Device testing demonstrates efficient electroluminescence (EL) performance, with maximum EQE values of 15.2% at 628 nm, 19.7% at 646 nm, and 6.1% at 686 nm. The (R/S)-OBN-3CN- and (R/S)-OBN-4CN-doped OLEDs exhibit significant circularly polarized electroluminescence (CPEL) activities, with the |g_EL_| can reach 1.1 × 10^−3^ and 1.0 × 10^−3^, respectively. Furthermore, Yao et al. designed a receptor incorporating naphthalene [1,2-b] pyrazine-8,9-dicarbonamide (AP) and combined it with triphenylamine and a chiral unit, octahydronaphthol, to construct a pair of highly efficient near-infrared emitting chiral enantiomers, (R/S)-ONTAP [48]. In doped films, these molecules achieve a fluorescence quantum yield of 100%, with a chiral luminescence asymmetry factor reaching the order of 10^−3^. The doped OLED exhibits a maximum EQE of 13.7% at 676 nm, while the undoped devices achieve a maximum EQE of 2.3% at 748 nm.

Xiang et al. developed a structure analogous to phenoxazine using chiral octahydronaphthol as both an electron donor and a chiral unit. This structure was connected to 2-(4-bromophenyl)-4,6-diphenyl-1,3,5-triazine to form the chiral enantiomer (R/S)-BIPNX-TRZ [49]. By creating a unique three-propeller structure, the molecule demonstrated exceptional TADF performance, achieving a fluorescence quantum yield of 57.6% under doping conditions. The corresponding chiral OLEDs exhibited good chiral luminescence ability, with a maximum EQE of 10.2% at an emission wavelength of 550 nm. Utilizing a semi-transparent structure, the g_EL_ was doubled, reaching approximately 1.69 × 10^−3^. This work adopts a semi-transparent device structure, which is an effective strategy to enhance the g_EL_ of CP-OLED devices.

Su et al. constructed chiral molecules, (R/S)-p-hex and (R/S)-o-hex (Figure 6), with both TADF and room-temperature phosphorescence (RTP) mechanisms using phenyldithiol, naphthol, and dicyanobenzene [39]. By simply changing the position of each unit, two molecules with different luminescent properties can be obtained. P-hex exhibits TADF properties and has good photoluminescence ability, with a fluorescence quantum yield of 94.4% in the thin film state. The corresponding emission peak of CP-OLED device is located at 515 nm, with the maximum EQE reaching 30.6%, and o-hex exhibits both TADF and RTP mechanisms, corresponding to warm white light, with the maximum EQE reaching 2.10%.

### 2.3. 1.2-Diaminocyclohexane

**Figure 7:**
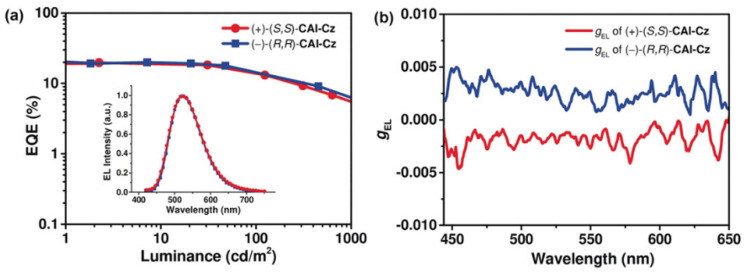
Performance of OLEDs based on (+)-(*S*,*S*)-CAI-Cz and (−)-(*R*,*R*)-CAI-Cz as emitters. (**a**) EQE–luminance characteristics. Inset: EL spectra of the devices at 9 V. (**b**) The *g*_EL_ value of the enantiomer-based OLEDs as a function of emission wavelength. Copyright 2018, Wiley-VCH [50].

**Figure 8:**
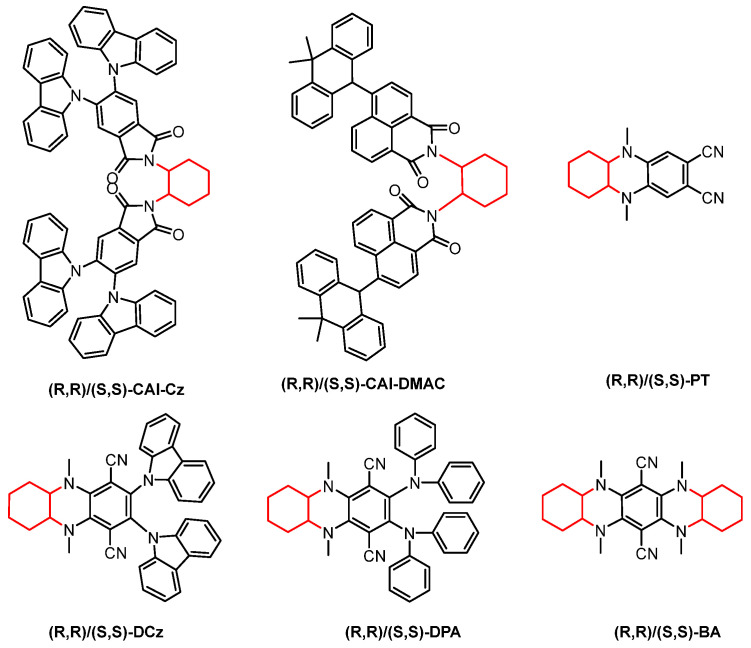
1.2-Diaminocyclohexane-based chiral CP-TADF molecules. (The red part in the picture represents the chiral unit).

In 2018, Chen et al. first reported a chiral TADF material, (R/S)-CAI-Cz,based on chiral 1,2-diaminocyclohexane [50]. This material exhibited excellent TADF properties with a PLQY of 98%. The enantiomers demonstrated mirror-like circular dichroism (CD) and CPL properties. The corresponding OLED device emitted at a peak wavelength of 520 nm, achieving maximum EQE values of 19.8% and 19.7%, respectively. During CP-OLED operation, the enantiomers exhibited opposite CPEL signals, with g_EL_ values of +2.3 × 10^−3^ and −1.7 × 10^−3^, respectively (Figure 7). Subsequently, they modified the electron donor to dimethylacridine, synthesizing a pair of enantiomers, (R/S)-CAI-DMAC (Figure 8) [51]. When doped into a CBP matrix, the material achieved a fluorescence quantum yield of 39.9% and a luminescence asymmetry factor (g_lum_) of ±9.2 × 10^−4^. OLED devices utilizing this molecule emitted at a peak wavelength of 592 nm, with maximum EQE values of 12.4% and 12.3%, respectively.

In 2020, Zheng et al. synthesized four enantiomers—(R,R)/(S,S)-BA, (R,R)/(S,S)-DCz, (R,R)/(S,S)-DPA, and (R,R)/(S,S)-PT—based on chiral 1,2-diaminocyclohexane (Figure 8) [52]. These compounds exhibited multi-color circularly polarized luminescence within the 420–610 nm range. In toluene solution, the PT series displayed the lowest PLQY (22.4%), while the DCz series demonstrated the highest PLQY (93.14%). These molecules were incorporated into OLED devices, where the integration of carbazole donors significantly enhanced device performance. The maximum EQE values for BA, DCz, DPA, and PT were 2.0%, 5.5%, 2.5%, and 1.7%, respectively. Additionally, these devices exhibited favorable electroluminescence performance, with an electroluminescence asymmetry factor |g_EL_| ranging from 4.5 × 10^−4^ to 1.4 × 10^−3^.

### 2.4. Other Chiral Units

**Figure 9:**
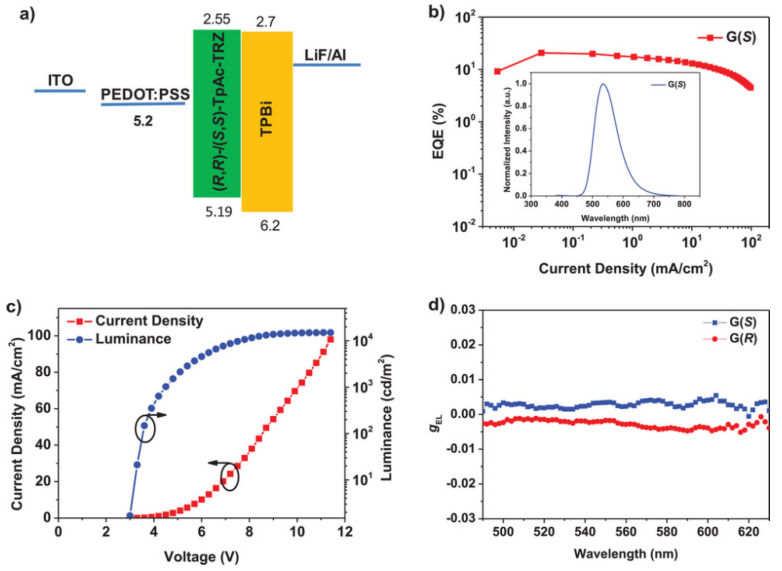
(**a**) Energy diagram of the solution-processed non-doped CP-OLEDs based on (*R*/*S*)-TpAc-TRZ. (**b**) EQE–current density characteristics of the device G(*S*). Inset: EL spectra of the device at 6 V. (**c**) Current density–voltage–luminance (*J*–*V–L*) characteristics of the device G(*S*). (**d**). g_EL_ values of CP-OLEDs based on (*S*)-(+)-TpAc-TRZ (G(*S*)) and (*R*)-(−)-TpAc-TRZ (G(*R*)) as a function of emission wavelength. Copyright 2021, Wiley-VCH [53].

The rigid structure of chiral units can mitigate the influence of molecular π-π stacking, which positively contributes to enhancing the luminescence efficiency of CP-TADF molecules in the aggregated state. Chen et al. utilized a rigid three-dimensional triazine structure to construct a pair of novel CP-TADF materials, (R/S)-TpAc-TRZ, achieving a PLQY of 85% in the thin film state [53]. When employed as the luminescent material for CP-OLED devices, these molecules exhibit a maximum emission peak at 534 nm, with a corresponding maximum EQE of 25.5%. Additionally, the devices demonstrate a favorable mirrored CPEL signal, with an electroluminescence asymmetry factor (g_EL_) of +1.5/−2.0 × 10^−3^. They further utilized triazine to develop another pair of novel CP-TADF materials, (R/S)-CTRI-Cz [54]. Upon doping thin films from air to vacuum state, the PLQY increased from 44% to 57%. Chiral studies revealed that their luminescence asymmetry factor (g_lum_) reaches ±0.9 × 10^−3^. When incorporated as OLED light-emitting layers, these materials exhibit an emission peak at 520 nm, achieving a maximum EQE of 15.0% (Figure 9).

**Figure 10:**
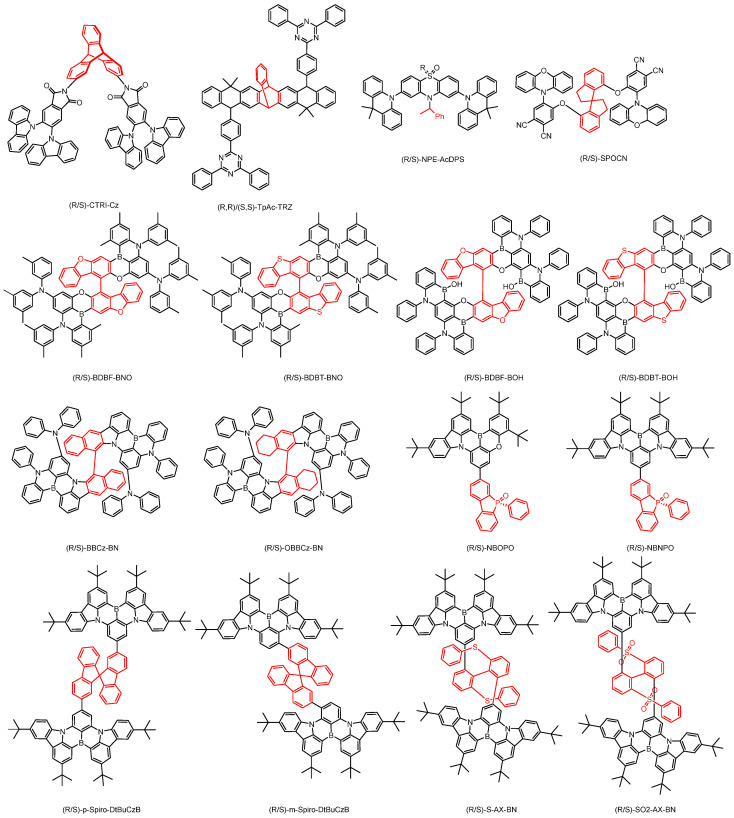
Chiral CP-TADF molecules constructed based on other chiral units. (The red part in the picture represents the chiral unit).

In 2021, Yang et al. introduced chiral (R/S)-1-phenylethylamine onto the excellent blue TADF molecule DMAC-DPS to construct a new CP-TADF molecule (R/S)-NPE-ACDPS (Figure 10) [55]. This enantiomer still maintains excellent TADF properties and has a high PLQY of 86%. Using this pair of ligands as the light-emitting layer of OLED, the maximum emission peak is located at 458 nm, with a maximum EQE of 18.5%.

Zheng et al. synthesized a pair of TADF enantiomers (R/S)-SOCN based on spirodiol, using ortho phenylenedicyanide as the acceptor unit and phenoxazine as the donor unit [56]. This enantiomer exhibits excellent TADF properties, with a very small ΔE_ST_ of 0.03 eV, a maximum PLQY of 69.4%, and a delayed-fluorescence lifetime of 5.0 µs. Using (R/S)-SPOCN as the emitting layer of OLED, a yellow emission at 566 nm was obtained, with maximum EQE values of 14.4% and 15.8%, and |g_EL_| values of 1.7 × 10^−3^, respectively. For the first time, it was reported that two CP-TADF molecules were used as complementary light to achieve white circularly polarized light emissions. They employed the previously reported CP-TADF molecule (R/S)-OSFSO as a blue emitter in a white-light-emitting system [57]. This was combined with the orange–red emitter (R/S)-SOCN in specific ratios to form an efficient hybrid white-light-emitting system. When fabricated into OLED devices, the system emitted white circularly polarized light with maximum EQE values of 20.4% and 19.7%, and the corresponding |g_EL_| values can reach 2.5 × 10^−3^ and −3.0 × 10^−3^, respectively.

Zheng et al. utilized (R/S)-(1,1′-bidibenzo[b,d]furan)-2,2′-diol and (R/S)-(1,1′-bidibenzo[b,d]thiophene)-2,2′-diol as chiral sources to construct two enantiomers, (R/S)-BDBF-BNO and (R/S)-BDBT-BNO (Figure 10), incorporating multiple resonance units [58]. The incorporation of chiral units into the distribution of frontier molecular orbitals (FMO) results in excellent photoluminescence properties for these molecules. In toluene solution, the maximum emission peak occurs at 460 nm with a half-peak width of 27 nm. The fluorescence quantum yields in doped films are as high as 93% and 96%, respectively. When employed as the emitting layer in OLEDs, BDBF-BNO exhibits blue electroluminescence with an emission peak at 473 nm and a half-peak width of 38 nm. The maximum EQE is 32.1%, with a corresponding |g_EL_| can reach 1.5 × 10^−3^ and 1.3 × 10^−3^. Interestingly, the introduction of sulfur atoms in BDBT-BNO enhances the spin–orbit coupling of the molecule, resulting in improved maximum EQE performance (35.7%), and |g_EL_| values can reach 1.5 × 10^−3^ and 1.6 × 10^−3^ for the corresponding OLED. In the same year, they further expanded the application range of the chiral unit on CP-MR-TADF molecules by preparing enantiomers (R/S)-BDBF-BOH and (R/S)-BDBT-BOH using the same chiral source [59]. These enantiomers also exhibit excellent photoluminescence properties, with a half-peak width of 27 nm and fluorescence quantum yields exceeding 90%. During OLED fabrication, the maximum emission peak is located at 467 nm, achieving maximum EQE values of 29.5% and 30.1%, respectively. The corresponding |g_EL_| values can reach 0.59 × 10^−3^ and 1.2 × 10^−3^, respectively. Through these two works, it was further confirmed that the participation of heavy atoms of S has a positive effect on improving the external quantum effects of the device, and the glum is also enhanced to a certain extent with the improvement of device efficiency.

Modulating the magnetic dipole moment (*θ*_e,m_) could be an effective strategy to enhance chiral luminescence properties. To explore this hypothesis, Zheng et al. employed (R/S)-5*H*,5′*H*-6,6′-bibenzo[b]carbazole and (R/S)-7,7′,8,8′,9,9′,10,10′-octahydro-5*H*,5′*H*-6,6′-bibenzo[b]carbazole as chiral sources to synthesize the enantiomers (R/S)-BBCz-BN and (R/S)-OBBCz-BN [60]. The high conjugation degree of (R/S)-5*H*,5′*H*-6,6′-bibenzo[b]carbazole reduces its electron-donating ability, resulting in (R/S)-BBCz-BN lacking TADF properties. In contrast, (R/S)-OBBCz-BN exhibits superior TADF properties due to the use of (R/S)-7,7′,8,8′,9,9′,10,10′-octahydro-5*H*,5′*H*-6,6′-bibenzo[b]carbazole, which has a lower conjugation degree. Theoretical calculations reveal that (R/S)-BBCz-BN has a smaller *θ*_e,m_ compared to (R/S)-OBBCz-BN. Chiral studies demonstrate that the luminescence asymmetry factor of (R/S)-BBCz-BN is approximately three times that of (R/S)-OBBCz-BN. Thus, a smaller *θ*_e,m_ positively influences the enhancement of chiral signals in chiral molecules. When used as luminescent materials in OLEDs, (R/S)-BBCz-BN and (R/S)-OBBCz-BN exhibit emission peaks at 490 nm and 480 nm, respectively, with a half-peak width of approximately 30 nm. The maximum EQE values are 24.7% and 33.3%, respectively, and corresponding |g_EL_| values can reach 3.4 × 10^−3^ and 1.2 × 10^−3^.

Zheng et al. utilized chiral 5-phenyl-5*H*-dibenzophosphole-5-oxide, containing a P = O unit, to prepare two pairs of enantiomers, (R/S)-NBOPO and (R/S)-NBNPO (Figure 10) [61]. These molecules exhibit excellent photoluminescence performance, with a half-peak width of approximately 25 nm and fluorescence quantum yields of about 90%. When employed as luminescent materials in OLEDs, NBOPO and NBNPO exhibit emission peaks at 468 nm and 502 nm, respectively, with half-peak widths of 31 nm and 29 nm. The maximum EQE values are 16.4% and 28.3%, respectively, with corresponding electroluminescence asymmetry factors |g_EL_| can reach 1.46 × 10^−3^ and 7.80 × 10^−4^. Additionally, they used chiral 9,9′-spirobi [9*H*-fluorene] as a chiral source and synthesized two pairs of enantiomers, (R/S)-p-spiro-DtBuCzB and (R/S)-m-spiro-DtBuCzB, by modifying the sites connected to the multi-resonance hosts [62]. These molecules retain the excellent photoluminescence properties of multi-resonance materials, with fluorescence quantum yields exceeding 95% and half-peak widths of 25 nm and 33 nm, respectively. OLED devices fabricated with these materials exhibit emission peaks at 498 nm and 508 nm, achieving maximum EQE values of 29.6% and 33.8%, respectively. The corresponding |g_EL_| values can reach 3.49 × 10^−4^ and 7.85 × 10^−4^. Furthermore, they incorporated biphenyl derivatives as chiral units and combined them with traditional multi-resonance (MR) molecules to prepare (R/S)-S-AX-BN and (R/S)-SO_2_-AX-BN [63]. The introduction of -Ph (thiobenzene) and -SO_2_Ph (sulfonylbenzene) groups onto the chiral units allows for fine-tuning of the emission wavelength. These enantiomers demonstrate outstanding luminescence performance, with fluorescence quantum yields exceeding 90% and extremely narrow half-peak widths (approximately 20 nm). During OLED fabrication, the devices retain a small half-peak width (about 21 nm), with emission peaks at 495 nm (EQE_max_ = 33.5%) and 500 nm (EQE_max_ = 31.5%). The devices exhibit good CPEL signals, with |g_EL_| values reaching 3.3 × 10^−3^ and 2.2 × 10^−3^, respectively.

## 3. Conclusions and Outlook

TADF materials are extensively utilized in display devices due to their thermal stability and exceptional PLQY. CP-TADF materials, which integrate TADF and CPL, effectively minimize energy loss during the luminescence process, thereby contributing to energy conservation. In this paper, we present a comprehensive review of CP-TADF small molecules developed through the chiral perturbation strategy. These molecules exhibit robust electroluminescence capabilities. The direct synthesis of CP-TADF molecules from existing chiral materials significantly reduces preparation costs, rendering it more conducive to industrial production. Nonetheless, this research remains in its nascent stages, necessitating further exploration and refinement by scientific investigators. While several outstanding CP-TADF molecules have been synthesized at this juncture, characterized by high PLQY, EQE, and g_lum_, certain challenging issues persist.

The majority of CP-TADF molecules constructed via the chiral perturbation strategy emit light within the 480 to 600 nm range, with limited examples of efficient blue (<480 nm) and red (>600 nm) emitters. A promising approach may involve the integration of highly efficient blue and red light-emitting TADF molecules with chiral counterparts through the chiral perturbation strategy. Additionally, the development of highly efficient OLED materials can be achieved by doping a chiral host with a TADF luminescent guest, provided the molecular design is meticulously planned.

To facilitate the industrialization of CP-OLEDs, it is imperative to develop devices that exhibit high EQE, low efficiency roll-off, high color purity, and extended operational lifespan. The TADF sensitization strategy and multiple resonance schemes offer potential solutions to these challenges. The TADF strategy can amplify the electroluminescence asymmetry factor by orders of magnitude, while boron–nitrogen (B-N) compounds can narrow the electroluminescence spectrum, thereby enhancing color purity.

## Data Availability

Data availability is not applicable to this article as no new datasets were generated or analyzed during the current study.

## References

[1] Chen F., Qiu C., Liu Z. (2022). Investigation of Autostereoscopic Displays Based on Various Display Technologies. Nanomaterials.

[2] Diesing S., Zhang L., Zysman-Colman E., Samuel I.D.W. (2024). A Figure of Merit for Efficiency Roll-off in TADF-Based Organic LEDs. Nature.

[3] Hong G., Gan X., Leonhardt C., Zhang Z., Seibert J., Busch J.M., Bräse S. (2021). A Brief History of OLEDs—Emitter Development and Industry Milestones. Adv. Mater..

[4] Siddiqui I., Kumar S., Tsai Y.-F., Gautam P., Shahnawaz, Kesavan K., Lin J.-T., Khai L., Chou K.-H., Choudhury A. (2023). Status and Challenges of Blue OLEDs: A Review. Nanomaterials.

[5] Fang H., Li J., Gong S., Lin J., Xie G. (2024). Inkjet Printing of High-Color-Purity Blue Organic Light-Emitting Diodes with Host-Free Inks. Molecules.

[6] Tang C.W., Vanslyke S.A. (1987). Organic Electroluminescent Diodes. Appl. Phys. Lett..

[7] Shin H., Lee S., Kim K., Moon C., Yoo S., Lee J., Kim J. (2014). Blue Phosphorescent Organic Light-Emitting Diodes Using an Exciplex Forming Co-host with the External Quantum Efficiency of Theoretical Limit. Adv. Mater..

[8] Li C., Duan C., Han C., Xu H. (2018). Secondary Acceptor Optimization for Full-Exciton Radiation: Toward Sky-Blue Thermally Activated Delayed Fluorescence Diodes with External Quantum Efficiency of ≈30%. Adv. Mater..

[9] Xu Y., Li C., Li Z., Wang Q., Cai X., Wei J., Wang Y. (2020). Constructing Charge-Transfer Excited States Based on Frontier Molecular Orbital Engineering: Narrowband Green Electroluminescence with High Color Purity and Efficiency. Angew. Chem. Int. Ed. Engl..

[10] Huang R., Chen H., Liu H., Zhuang Z., Wang J., Yu M., Yang D., Ma D., Zhao Z., Tang B.Z. (2022). Creating Efficient Delayed Fluorescence Luminogens with Acridine-Based Spiro Donors to Improve Horizontal Dipole Orientation for High-performance OLEDs. Chem. Eng. J..

[11] Yang S., Qu Y., Liao L., Jiang Z., Lee S. (2021). Research Progress of Intramolecular π-Stacked Small Molecules for Device Applications. Adv. Mater..

[12] Rui J., Pu J., Chen Z., Tang H., Wang L., Su S.-J., Cao D. (2024). Single-molecule White Organic Light-Emitting Diodes Based on Dual-Conformation Diindolophenthiazine Derivatives. J. Mater. Chem. C.

[13] Jang E.-B., Choi G.-S., Bae E.-J., Ju B.-K., Park Y.-W. (2023). Doping-Free Phosphorescent and Thermally Activated Delayed Fluorescent Organic Light-Emitting Diodes with an Ultra-Thin Emission Layer. Nanomaterials.

[14] Wu Z.-L., Lv X., Meng L.-Y., Chen X.-L., Lu C.-Z. (2023). Tröger’s Base-Derived Thermally Activated Delayed Fluorescence Dopant for Efficient Deep-Blue Organic Light-Emitting Diodes. Molecules.

[15] Zhang Q., Li J., Shizu K., Huang S., Hirata S., Miyazaki H., Adachi C. (2012). Design of Efficient Thermally Activated Delayed Fluorescence Materials for Pure Blue Organic Light Emitting Diodes. J. Am. Chem. Soc..

[16] Wang Z., Qu C., Liang J., Zhuang X., Liu Y., Wang Y. (2024). High-Efficiency and Narrowband Green Thermally Activated Delayed Fluorescence Organic Light-Emitting Diodes Based on Two Diverse Boron Multi-Resonant Skeletons. Molecules.

[17] Deng Y., Wang M., Zhuang Y., Liu S., Huang W., Zhao Q. (2021). Circularly Polarized Luminescence from Organic Micro-/Nano-Structures. Light. Sci. Appl..

[18] Li X., Xie Y., Li Z. (2020). The Progress of Circularly Polarized Luminescence in Chiral Purely Organic Materials. Adv. Photon- Res..

[19] Gong Z., Li Z., Zhong Y. (2022). Circularly Polarized Luminescence of Coordination Aggregates. Aggregate.

[20] Imai Y., Amasaki R., Yanagibashi Y., Suzuki S., Shikura R., Yagi S. (2024). Magnetically Induced Near-Infrared Circularly Polarized Electroluminescence from an Achiral Perovskite Light-Emitting Diode. Magnetochemistry.

[21] Zhao W., Tan K., Guo W., Guo C., Li M., Chen C. (2024). Acceptor Copolymerized Axially Chiral Conjugated Polymers with TADF Properties for Efficient Circularly Polarized Electroluminescence. Adv. Sci..

[22] Yang Y., Xiao S., Zhou Y., Shi C., Xu L., Liao X., Sun N., Zheng Y.-X., Ding L., Ding J. (2024). Chiral perturbation in D-O-A organic Phosphors towards Efficient Circularly Polarized Electroluminescence. Sci. China Mater..

[23] Wang Y., Li M., Teng J., Zhou H., Zhao W., Chen C. (2021). Chiral TADF-Active Polymers for High-Efficiency Circularly Polarized Organic Light-Emitting Diodes. Angew. Chem. Int. Ed. Engl..

[24] Zhong X., Xi J., Yan Z., Hu J., Wang Y., Yuan L., Song S., Zuo J., Zheng Y. (2025). Efficient Circularly Polarized Electroluminescence Based on Chiral MR-TADF Materials with Innovative Planar Structure Design Strategy. Adv. Funct. Mater..

[25] Imagawa T., Hirata S., Totani K., Watanabe T., Vacha M. (2015). Thermally Activated Delayed Fluorescence with Circularly Polarized Luminescence Characteristics. Chem. Commun..

[26] Feuillastre S., Pauton M., Gao L., Desmarchelier A., Riives A.J., Prim D., Tondelier D., Geffroy B., Muller G., Clavier G. (2016). Design and Synthesis of New Circularly Polarized Thermally Activated Delayed Fluorescence Emitters. J. Am. Chem. Soc..

[27] Song F., Xu Z., Zhang Q., Zhao Z., Zhang H., Zhao W., Qiu Z., Qi C., Zhang H., Sung H.H.Y. (2018). Highly Efficient Circularly Polarized Electroluminescence from Aggregation-Induced Emission Luminogens with Amplified Chirality and Delayed Fluorescence. Adv. Funct. Mater..

[28] Sun S., Wang J., Chen L., Chen R., Jin J., Chen C., Chen S., Xie G., Zheng C., Huang W. (2019). Thermally Activated Delayed Fluorescence Enantiomers for Solution-Processed Circularly Polarized Electroluminescence. J. Mater. Chem. C.

[29] Frédéric L., Desmarchelier A., Plais R., Lavnevich L., Muller G., Villafuerte C., Clavier G., Quesnel E., Racine B., Meunier-Della-Gatta S. (2020). Maximizing Chiral Perturbation on Thermally Activated Delayed Fluorescence Emitters and Elaboration of the First Top-Emission Circularly Polarized OLED. Adv. Funct. Mater..

[30] Yan Z.P., Liu T.T., Wu R., Liang X., Li Z.Q., Zhou L., Zheng Y.X., Zuo J.L. (2021). Chiral Thermally Activated Delayed Fluorescence Materials Based on R/S-N2,N2′-Diphenyl-[1,1′-binaphthalene]-2,2′-diamine Donor with Narrow Emission Spectra for Highly Efficient Circularly Polarized Electroluminescence. Adv. Funct. Mater..

[31] Liang J., Hu J., Huo Z., Yan Z., Yuan L., Zhong X., Wei Y., Song S., Liu Q., Song Y. (2024). Two Different Chiral Groups Based Thermally Activated Delayed Fluorescence Materials for Circularly Polarized OLEDs. Chem.-Asian J..

[32] Zhang Y., Li J., Quan Y., Ye S., Cheng Y. (2020). Solution-Processed White Circularly Polarized Organic Light-Emitting Diodes Based on Chiral Binaphthyl Emitters. Chem.-A Eur. J..

[33] Xue P., Wang X., Wang W., Zhang J., Wang Z., Jin J., Zheng C., Li P., Xie G., Chen R. (2021). Solution-Processable Chiral Boron Complexes for Circularly Polarized Red Thermally Activated Delayed Fluorescent Devices. ACS Appl. Mater. Interfaces.

[34] Zhou L., Ni F., Li N., Wang K., Xie G., Yang C. (2022). Tetracoordinate Boron-Based Multifunctional Chiral Thermally Activated Delayed Fluorescence Emitters. Angew. Chem. Int. Ed. Engl..

[35] Zhu L., Liu D., Wu K., Xie G., Zhao Z., Tang B.Z. (2024). Delayed Fluorescence and Amplified Chirality via Modified Substitution Position for Deep-red Circularly Polarized Organic Light Emitting-diodes. Chem. Res. Chin. Univ..

[36] Tong J., Wang P., Liao X., Wang Y., Zheng Y., Pan Y. (2024). Chiral Sulfonyl Binaphthalene-Based Thermally Activated Delayed Fluorescence Materials for Circularly Polarized Electroluminescence. Adv. Opt. Mater..

[37] Xing S., Zhong X., Liao X., Wang Y., Yuan L., Ni H., Zheng Y. (2024). Axially Chiral Multiple Resonance Thermally Activated Delayed Fluorescence Enantiomers for Efficient Circularly Polarized Electroluminescence. Adv. Opt. Mater..

[38] Gong M., Guo X., Yuan L., Jiang H., Zeng G., Zheng W.-H., Zheng Y.-X. (2025). Axially Chiral Biphenoxazine-Based Multi-Resonance Thermally Activated Delayed Fluorescence Materials for Circularly Polarized Electroluminescence. Chem. Eng. J..

[39] Li M., Li Z., Peng X., Liu D., Chen Z., Xie W., Liu K., Su S. (2025). Excited-State Engineering of Chalcogen-Bridged Chiral Molecules for Efficient OLEDs with Diverse Luminescence Mechanisms. Angew. Chem. Int. Ed. Engl..

[40] Wu Z.-G., Yan Z.-P., Luo X.-F., Yuan L., Liang W.-Q., Wang Y., Zheng Y.-X., Zuo J.-L., Pan Y. (2019). Non-Doped and Doped Circularly Polarized Organic Light-Emitting Diodes with High Performances Based on Chiral Octahydro-Binaphthyl Delayed Fluorescent Luminophores. J. Mater. Chem. C.

[41] Wu Z., Han H., Yan Z., Luo X., Wang Y., Zheng Y., Zuo J., Pan Y. (2019). Chiral Octahydro-Binaphthol Compound-Based Thermally Activated Delayed Fluorescence Materials for Circularly Polarized Electroluminescence with Superior EQE of 32.6% and Extremely Low Efficiency Roll-Off. Adv. Mater..

[42] Xie F., Zhou J., Zeng X., An Z., Li Y., Han D., Duan P., Wu Z., Zheng Y., Tang J. (2021). Efficient Circularly Polarized Electroluminescence from Chiral Thermally Activated Delayed Fluorescence Emitters Featuring Symmetrical and Rigid Coplanar Acceptors. Adv. Opt. Mater..

[43] Xu Y., Wang Q., Cai X., Li C., Wang Y. (2021). Highly Efficient Electroluminescence from Narrowband Green Circularly Polarized Multiple Resonance Thermally Activated Delayed Fluorescence Enantiomers. Adv. Mater..

[44] Wang Y.-F., Liu X., Zhu Y., Li M., Chen C.-F. (2022). Aromatic-Imide-Based TADF Enantiomers for Efficient Circularly Polarized Electroluminescence. J. Mater. Chem. C.

[45] Liu T.-T., Yan Z.-P., Hu J.-J., Yuan L., Luo X.-F., Tu Z.-L., Zheng Y.-X. (2021). Chiral Thermally Activated Delayed Fluorescence Emitters-Based Efficient Circularly Polarized Organic Light-Emitting Diodes Featuring Low Efficiency Roll-Off. ACS Appl. Mater. Interfaces.

[46] Yan Z., Yuan L., Zhang Y., Mao M., Liao X., Ni H., Wang Z., An Z., Zheng Y., Zuo J. (2022). A Chiral Dual-Core Organoboron Structure Realizes Dual-Channel Enhanced Ultrapure Blue Emission and Highly Efficient Circularly Polarized Electroluminescence. Adv. Mater..

[47] Sun B., Ding L., Wang X., Tu Z.-L., Fan J. (2023). Circularly Polarized Thermally Activated Delayed Fluorescence OLEDs with Nearly BT.2020 Red Emission. Chem. Eng. J..

[48] Liang N., Liu J., Lin Y., Xie Z., Cui B., Gong Z., Gan Q., Zhong Y., Feng Y., Yao C. (2024). Construction of Circular Polarized Luminescence Molecules for Intense Near Infrared OLEDs. Adv. Opt. Mater..

[49] Qu L., Xiao H., Zhang B., Yang Q., Song J., Zhou X., Xu Z.-X., Xiang H. (2023). Axially Chiral Biphenoxazine-Based Thermally Activated Delayed Fluorescence Materials for Solution-Processed Circularly Polarized Organic Light-Emitting Diodes. Chem. Eng. J..

[50] Li M., Li S., Zhang D., Cai M., Duan L., Fung M., Chen C. (2018). Stable Enantiomers Displaying Thermally Activated Delayed Fluorescence: Efficient OLEDs with Circularly Polarized Electroluminescence. Angew. Chem. Int. Ed. Engl..

[51] Wang Y.-F., Lu H.-Y., Chen C., Li M., Chen C.-F. (2019). 1,8-Naphthalimide-Based Circularly Polarized TADF Enantiomers as The Emitters for Efficient Orange-Red OLEDs. Org. Electron..

[52] Luo X.-F., Han H.-B., Yan Z.-P., Wu Z.-G., Su J., Zou J.-W., Zhu Z.-Q., Zheng Y.-X., Zuo J.-L. (2020). Multicolor Circularly Polarized Photoluminescence and Electroluminescence with 1,2-Diaminecyclohexane Enantiomers. ACS Appl. Mater. Interfaces.

[53] Wang Y., Li M., Teng J., Zhou H., Chen C. (2021). High-Performance Solution-Processed Nondoped Circularly Polarized OLEDs with Chiral Triptycene Scaffold-Based TADF Emitters Realizing Over 20% External Quantum Efficiency. Adv. Funct. Mater..

[54] Wang Y.-F., Chen C., Cui L., Teng J.-M., Li M., Lu H.-Y., Chen C.-F. (2021). Triptycene-Derived TADF Enantiomers Displaying Circularly Polarized Luminescence and High-efficiency Electroluminescence. Org. Electron..

[55] Huang Z., Huang C.-W., Tang Y.-K., Xiao Z., Li N., Hua T., Cao X., Zhou C., Wu C.-C., Yang C. (2022). Chiral Thermally Activated Delayed Fluorescence Emitters for Circularly Polarized Luminescence and Efficient Deep Blue OLEDs. Dye. Pigment..

[56] Zhang Y., Mao M., Song S., Wang Y., Zheng Y., Zuo J., Pan Y. (2022). Circularly Polarized White Organic Light-Emitting Diodes Based on Spiro-Type Thermally Activated Delayed Fluorescence Materials. Angew. Chem. Int. Ed. Engl..

[57] Zhang Y., Liang X., Luo X., Song S., Li S., Wang Y., Mao Z., Xu W., Zheng Y., Zuo J. (2021). Chiral Spiro-Axis Induced Blue Thermally Activated Delayed Fluorescence Material for Efficient Circularly Polarized OLEDs with Low Efficiency Roll-Off. Angew. Chem. Int. Ed. Engl..

[58] Yuan L., Yang Y., Yan Z., Hu J., Mao D., Ni H., Zheng Y. (2024). Circularly Polarized Electroluminescence from Intrinsically Axial Chiral Materials Based on Bidibenzo[*b,d*]furan/bidibenzo[*b,d*]thiophene. Adv. Funct. Mater..

[59] Yuan L., Xu J., Yan Z., Yang Y., Mao D., Hu J., Ni H., Li C., Zuo J., Zheng Y. (2024). Tetraborated Intrinsically Axial Chiral Multi-resonance Thermally Activated Delayed Fluorescence Materials. Angew. Chem. Int. Ed. Engl..

[60] Yuan L., Mao D., Yan Z., Hu J., Ni H., Hong X., Zuo J., Zheng Y. (2025). Controlling of Circularly Polarized Luminescence via Modulating the Angle Between Transition Electric and Magnetic Dipole Moments. Adv. Funct. Mater..

[61] Wang Y., Lv Z.-Y., Chen Z.-X., Xing S., Huo Z.-Z., Hong X.-F., Yuan L., Li W., Zheng Y.-X. (2024). Multiple-Resonance Thermally Activated Delayed Fluorescence Materials Based on Phosphorus Central Chirality for Efficient Circularly Polarized Electroluminescence. Mater. Horizons.

[62] Song S.-Q., Han X., Huo Z.-Z., Yip C.-F., Hong X.-F., Ding M.-N., Zheng Y.-X. (2024). Chiral Multiple-Resonance Thermally Activated Delayed Fluorescence Materials Based on Chiral Spiro-Axis Skeleton for Efficient Circularly Polarized Electroluminescence. Sci. China Chem..

[63] Wang X., Xing S., Xiao X., Yuan L., Hou Z., Zheng Y. (2024). Axial Chiral Biphenyl MR-TADF Enantiomers for Efficient Narrowband Circularly Polarized Electroluminescence. Adv. Funct. Mater..

